# Supplementary material for: Identification of photoperiod-induced specific miRNAs in the adrenal glands of Sunite sheep (Ovis aries)
Source: Front Vet Sci. 2022 Jul 22;9:888207. doi: 10.3389/fvets.2022.888207 (PMC9354845; doi:10.3389/fvets.2022.888207)
Supplement: Supplementary file 1 [file Data_Sheet_1.ZIP › Supplementary file/miRDeep2 file.zip!/miRDeep2 file/Know_Structure/LP42A3/oar-mir-665.pdf]

miRBase precursor : oar-mir-665
Total read count : 112
oar-miR-665-5p read count : 1
oar-miR-665-3p read count : 104
remaining reads : 7

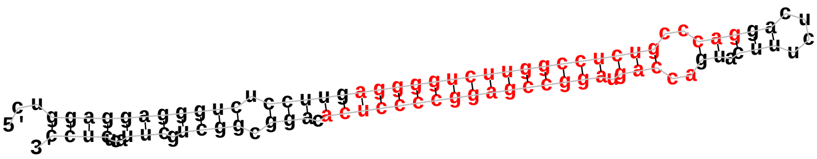

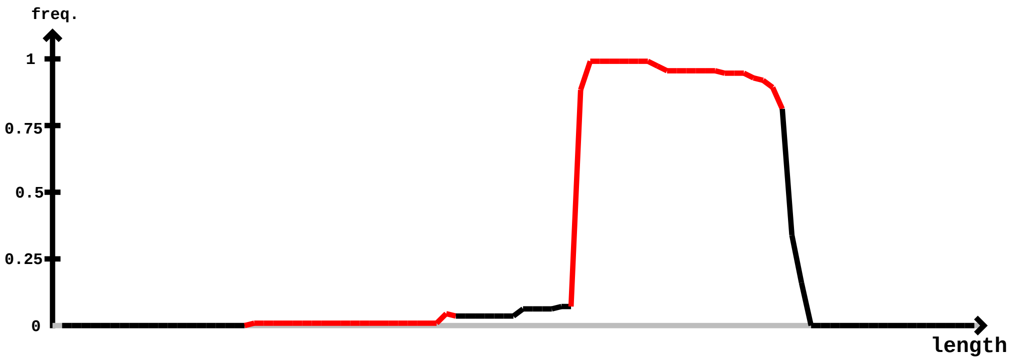


```
                                                      oar-miR-665-3p
                        oar-miR-665-5p
5'- cuggaggagggucuccuugaggggucuuggccucugcccaggacucuuucaugaccaguaggccgaggccccucacaggcggcugcuuacucucc   -3'    exp
    ..((((((((((.((((((((((((((((((((((..(((((....))).))..))).)))))))))))))))).))).)))).))).))))..   reads    mm     sample
    ...................aggggucuuggccucugccca.......................................................   1        0      seq
    .......................................aggacucuuucaugaccaguag..................................   2        0      seq
    .......................................aggacucuuucaugaccaguagg.................................   2        0      seq
    ...............................................uucaugaccaguaggccgagg...........................   1        0      seq
    ...............................................uucaugaccaguaggccgaggccc........................   2        0      seq
    ...................................................ugaccaguaggccgaggccccuc.....................   1        0      seq
    .....................................................accaguaggccgaggcccc.......................   1        0      seq
    .....................................................accaguaggccgaggccccu......................   3        0      seq
    .....................................................accaguaggccgaggccccuc.....................   8        0      seq
    .....................................................accaguaggccgaggccccuca....................   52       0      seq
    .....................................................accaguaggccgaggccccucac...................   16       0      seq
    .....................................................accaguaggccgaggccccucaca..................   11       0      seq
    ......................................................ccaguaggccgaggccccuca....................   1        0      seq
    ......................................................ccaguaggccgaggccccucac...................   4        0      seq
    ......................................................ccaguaggccgaggccccucaca..................   7        0      seq
```
